# Intrathecal IgG synthesis as a biomarker for CNS involvement in anti-GQ1b antibody syndrome: a case report and hypothesis-generating treatment framework

**DOI:** 10.3389/fimmu.2026.1794662

**Published:** 2026-09-01

**Authors:** Han Zhou, Xiuming Guo

**Affiliations:** Department of Neurology, The First Affiliated Hospital of Chongqing Medical University, Chongqing, China

**Keywords:** anti-GQ1b antibody, Bickerstaff brainstem encephalitis, cerebrospinal fluid biomarkers, Guillain-Barré syndrome, intrathecal IgG synthesis, oligoclonal bands

## Abstract

Anti-GQ1b antibody syndromes span a clinical continuum from peripheral Guillain-Barré syndrome (GBS) to central Bickerstaff brainstem encephalitis (BBE), but reliable biomarkers of central nervous system (CNS) involvement that could guide treatment intensity are lacking. We report a 37-year-old woman with a relapsing GBS–Miller Fisher syndrome (MFS)–BBE overlap phenotype. In the first episode (March 2024), plasma exchange, intravenous methylprednisolone and IVIG were administered after anti-GD1a IgG was detected. After self-discontinuation of immunotherapy, she was readmitted in May 2024 with a two-month course of progressive weakness acutely worsened over four days, ophthalmoplegia, dysarthria, intermittent choking and bilateral extensor plantar responses. CSF showed albuminocytologic dissociation (protein 2.21 g/L; total cell count 40×10^6^/L with a normal nucleated cell count) together with evidence of intrathecal IgG synthesis (IgG index 1.13; synthesis rate 147.10 mg/24h; oligoclonal bands type III with homologous serum bands). Electrophysiology revealed an acute motor axonal neuropathy pattern. IVIG and methylprednisolone were administered, with substantial improvement but persistent mild dysarthria and ataxia at three-month follow-up. The diagnosis was based mainly on the clinical course and electrophysiological findings, supported by serology and CSF analysis. This case shows that intrathecal IgG synthesis may complement albuminocytologic dissociation as a marker of CNS involvement in anti-GQ1b syndromes and supports the hypothesis—requiring prospective testing—that CSF immunological profiling could inform treatment intensity.

## 1 Introduction

Anti-GQ1b antibody syndromes encompass a heterogeneous spectrum ranging from purely peripheral Miller Fisher syndrome (MFS) to centrally predominant Bickerstaff brainstem encephalitis (BBE), with classic Guillain-Barré syndrome (GBS) occupying an intermediate position (1–4). While phenotypic overlap is increasingly recognized, a critical unmet clinical need persists: how can clinicians reliably identify patients with significant CNS involvement who may benefit from intensified immunotherapy?

Current diagnostic paradigms rely heavily on clinical features and standard CSF findings, particularly albuminocytologic dissociation. However, elevated CSF protein with normal cell counts is ubiquitous across the anti-GQ1b spectrum and fails to discriminate peripheral-only disease from cases with true CNS inflammation (3, 5). This limitation has significant therapeutic implications, as standard GBS treatment trials typically excluded patients with overt CNS involvement (6–8).

Emerging evidence suggests that intrathecal IgG synthesis—quantified by an elevated IgG index, an increased synthesis rate, and CSF oligoclonal bands (OCBs)—may serve as a more specific biomarker for compartmentalized, antigen-driven B-cell activity within the CNS (9–12). Unlike simple protein elevation reflecting blood-brain barrier dysfunction, intrathecal synthesis implies active local immune responses that may be inadequately addressed by peripherally administered immunotherapy alone (9, 12). However, systematic data on intrathecal synthesis across the anti-GQ1b spectrum are scarce; OCBs have been documented in individual cases of BBE (13), and the clinical relevance of these markers for treatment decisions remains undefined. We present a case demonstrating how such CSF biomarkers can be integrated into therapeutic reasoning in a relapsing GBS–MFS–BBE overlap phenotype, and we propose— as a hypothesis—a biomarker-guided treatment framework.

## 2 Case presentation

### 2.1 First episode (March–April 2024)

**Figure 1:**
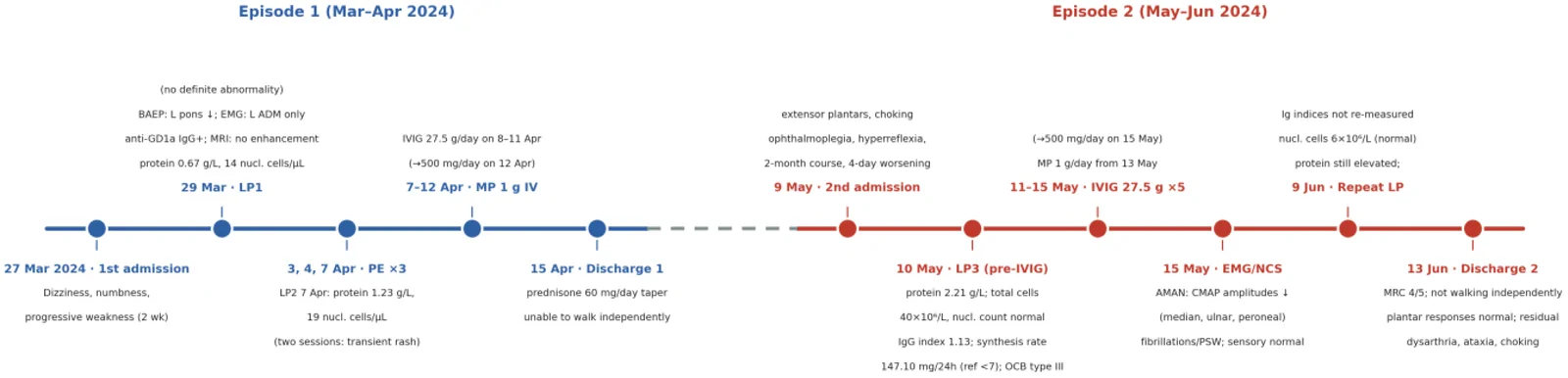
Clinical timeline of the two episodes. LP, lumbar puncture; OCB, oligoclonal bands; AMAN, acute motor axonal neuropathy; BAEP, brainstem auditory evoked potentials; EMG, electromyography; IVIG, intravenous immunoglobulin.

**Figure 1** summarizes the clinical timeline of both episodes. A 37-year-old woman with no significant medical history (past medical, family and psychosocial history were unremarkable, with no relevant comorbidities, regular medications, allergies, or smoking/alcohol use) presented on 27 March 2024 with a two-week history of episodic dizziness (“cotton-wool” sensation; each episode lasting ~30 s and recurring many times daily), transient stabbing ear pain, and numbness spreading from the right upper arm to the right trunk and left limbs, followed by progressive generalized weakness (difficulty raising the head, speaking and getting out of bed). A transient episode of hypotension (60/30 mmHg) had been resolved spontaneously. No preceding infection was reported. Examination showed clear consciousness with effortful, dysarthric speech; bilateral eyelid weakness; horizontal nystagmus with a full range of eye movements; a reduced gag reflex; MRC grade 4–5/5 weakness (right upper limb 4/5, elsewhere 5−/5); depressed tendon reflexes (particularly the ankle jerks); a questionably positive left Babinski sign; mildly impaired finger-to-nose testing; and reduced pinprick sensation on the right side.

Routine laboratory tests, ECG and extensive screening (ANA, ANCA, ASO, RF, HIV, syphilis, hepatitis B/C serology, HSV-1/2 DNA, CMV DNA, CSF culture, SARS-CoV-2 and respiratory virus nucleic acids) were unremarkable, except for a mildly elevated ESR (30 mm/h), polyclonal hypergammaglobulinemia (γ-globulin 23.1% on serum protein electrophoresis), an elevated D-dimer (2.24–2.62 mg/L FEU) and mildly reduced serum sodium. Serum anti-ganglioside antibody testing was positive for anti-GD1a IgG. CSF on 29 March showed protein 0.67 g/L (reference <0.45 g/L) with 14 nucleated cells/μL (total cell count 108×10^6^/L; Pandy weakly positive); glucose and chloride were normal and cultures were negative; CSF immunoglobulins and oligoclonal bands were not measured at that time.

Brain MRI on 28 March showed minor non-specific T2 hyperintensities in both frontoparietal regions (Fazekas grade I) with no enhancement; head-and-neck MRA showed no large-vessel stenosis, and the contrast-enhanced brain and spinal MRI on 29 March showed no pathological enhancement or leptomeningeal disease. Electrophysiology on 29 March showed a brainstem auditory evoked potential abnormality (reduced electrical activity in the left upper pons) with normal somatosensory and visual evoked potentials; needle EMG was limited to the left abductor digiti minimi (the patient declined further sampling) and showed no definite neurogenic or myogenic abnormality.

The patient was treated with three sessions of plasma exchange (3, 4 and 7 April 2024; two sessions were complicated by a transient pruritic rash treated with dexamethasone and promethazine), intravenous methylprednisolone 1 g/day from 7 April (reduced to 500 mg/day on 12 April), and IVIG 27.5 g/day on 8–11 April, followed by oral prednisone 60 mg/day with gradual taper. A repeat lumbar puncture on 7 April showed rising CSF protein (1.23 g/L; glucose 4.0 mmol/L) with 19 nucleated cells/μL (total cell count 1029×10^6^/L, likely reflecting blood admixture). Muscle strength improved modestly, and she was discharged on 15 April 2024 with residual limb numbness and inability to walk independently.

### 2.2 Second episode (May–June 2024): CSF immunological profiling

After discharge, the patient discontinued all immunomodulatory medication herself. Over the following weeks, weakness and numbness recurred and worsened acutely over four days, with new-onset intermittent choking on drinking, and she was readmitted on 9 May 2024 (cumulative symptomatic course approximately two months). Examination now showed moderate dysarthria; bilateral ophthalmoplegia with horizontal nystagmus; bilateral eyelid weakness; increased limb tone with right-sided MRC grade 4/5 and left-sided 3/5 weakness; brisk (3+) tendon reflexes; bilateral extensor plantar responses; and limb ataxia, with inability to walk independently. Admission laboratory tests showed hypokalemia (K 3.1–3.4 mmol/L), mildly reduced albumin (32 g/L) and elevated D-dimer (2.62 mg/L FEU).

Lumbar puncture on 10 May 2024, performed before the resumption of immunotherapy, revealed an opening pressure of ~110 mm H_2_O, protein 2.21 g/L (reference <0.45 g/L), total cell count 40×10^6^/L with a normal nucleated cell count (reference 0–8×10^6^/L), glucose 3.44 mmol/L and chloride 125 mmol/L—classic albuminocytologic dissociation. CSF immunological profiling demonstrated marked intrathecal IgG synthesis: IgG index 1.13 (reference ≤0.70), intrathecal IgG synthesis rate 147.10 mg/24h (reference <7 mg/24h; note that reference ranges vary across laboratories), and oligoclonal bands type III (CSF-specific bands with homologous serum bands on isoelectric focusing), a pattern consistent with intrathecal IgG synthesis in the setting of blood–CSF barrier disruption. CSF albumin was markedly elevated (1070.00 mg/L) and CSF IgG (47.00 mg/L), IgA (53.10 mg/L) and IgM (92.00 mg/L) were all increased, with elevated QAlb, QIgG, QIgA and QIgM quotients confirming severe blood–CSF barrier dysfunction. Because severe barrier dysfunction and systemic immune activation can also influence CSF immunoglobulin indices, these markers were interpreted as supportive—rather than definitive—evidence of compartmentalized CNS immune activation.

Repeated serology on the same date showed strongly positive anti-GQ1b IgG and positive anti-sulfatide IgG; anti-GD1a IgG remained positive. AQP4, MOG, GFAP and MBP antibodies and a six-item autoimmune encephalitis panel (NMDA, AMPA1/2, LGI1, GABAB, CASPR2) were negative in CSF and serum. A follow-up panel in June 2024 showed only weakly positive anti-GD1a and anti-sulfatide IgG, with anti-GQ1b no longer detectable, consistent with a post-treatment decline. Video-EEG on 11 May was normal. Nerve conduction studies on 15 May showed normal sensory conduction in the median, ulnar, radial, peroneal and sural nerves; markedly reduced compound muscle action potential amplitudes of the bilateral median, ulnar and right peroneal motor nerves (more severe on the left); reduced F-wave persistence of the bilateral ulnar nerves; and needle EMG showing fibrillation potentials and positive sharp waves in the bilateral tibialis anterior and abductor pollicis brevis muscles and the left biceps, with normal motor unit potential parameters and reduced recruitment—an acute motor axonal neuropathy (AMAN) pattern (14). Chest CT and abdominal ultrasound performed for paraneoplastic screening were unremarkable (incidental thyroid nodule and a small hepatic cyst). Nodal and paranodal antibodies (e.g., anti-neurofascin-155, anti-contactin-1, anti-CASPR1) were not tested. CSF smear microscopy was normal, and no leptomeningeal disease was identified on MRI.

Based on the clinical picture of a GBS–MFS–BBE overlap phenotype together with the CSF evidence of intrathecal IgG synthesis, IVIG 27.5 g/day was administered on 11–15 May 2024, and intravenous methylprednisolone 1 g/day was started on 13 May (reduced to 500 mg/day on 15 May and then stepwise to 80 mg before conversion to oral prednisone 55–60 mg/day with weekly 5 mg tapering), followed by continued rehabilitation. The patient was discharged on 13 June 2024 with improved strength (MRC 4/5 in all limbs) but persistent central deficits; the bilateral extensor plantar responses had converted to normal by discharge. Key diagnostic findings are summarized in **Table 1**.

**Table 1:** Summary of key diagnostic findings.

Investigation	Result	Reference range	Interpretation
Serum anti-GQ1b IgG (10 May)	Strongly positive	Negative	Anti-GQ1b antibody syndrome
Serum anti-GD1a IgG	Positive (Mar); weakly positive (Jun)	Negative	Supports axonal (AMAN) phenotype
Serum/CSF anti-sulfatide IgG	Positive (May); weakly positive (Jun)	Negative	Non-specific; reported in peripheral neuropathies
CSF (10 May 2024): protein	2.21 g/L	<0.45 g/L	Albuminocytologic dissociation
CSF total cells/nucleated cells	40×10^6^/L/normal	0–8×10^6^/L	Normal nucleated count
CSF glucose	3.44 mmol/L	2.2–3.9 mmol/L	Normal
IgG index	1.13	≤0.70	Suggestive of intrathecal synthesis*
IgG synthesis rate	147.10 mg/24h	<7 mg/24h	Markedly elevated (~21× upper limit)*
CSF albumin/IgG/IgA/IgM	1070.00/47.00/53.10/92.00 mg/L	—	Elevated; QAlb, QIgG, QIgA, QIgM quotients increased
Oligoclonal bands (isoelectric focusing)	Type III (CSF-specific bands with homologous serum bands)	Absent	Intrathecal IgG synthesis with blood–CSF barrier disruption
Motor nerve conduction (15 May)	Sensory normal; CMAP amplitudes markedly reduced (median, ulnar, peroneal); reduced ulnar F-wave persistence; fibrillations/PSW on needle EMG	—	Axonal injury (AMAN pattern)
Video-EEG (11 May)	Normal	—	No epileptiform activity

*Linear measures may overestimate intrathecal synthesis in the presence of severe blood–CSF barrier dysfunction; quotient (Reiber) analysis is the reference method in this setting, and the elevated QAlb and QIgG quotients confirmed both barrier dysfunction and intrathecal immunoglobulin production. Serum IgG and albumin values were not available for calculation of the Reiber intrathecal IgG fraction.

### 2.3 Outcome and follow-up

Clinician-assessed outcomes at defined time points showed progressive improvement in limb strength, speech and gait, but the patient could not walk independently at discharge and was transferred for ongoing rehabilitation. A repeat lumbar puncture on 9 June 2024 showed persistent protein elevation (Pandy 1+) with a normal nucleated cell count (6×10^6^/L); CSF immunoglobulin indices were not re-measured. At the three-month follow-up, limb strength was MRC 5/5 in the upper limbs and 4+/5 in the lower limbs, and she was living independently and working. Consistent with the initial evidence of CNS involvement, mild residual central deficits persisted: mild dysarthria (improved but not fully resolved), subtle truncal ataxia and occasional diplopia with sustained lateral gaze.

Patient perspective. The patient reported progressive recovery of daily function and work capacity and a gradual decrease in psychological burden as strength improved, and she returned to work by the three-month follow-up.

Adherence and adverse events. The patient discontinued all immunotherapy after the first discharge, which was followed by clinical relapse; adherence during the second episode was good. Treatment was generally well tolerated: two plasma-exchange sessions were complicated by a transient pruritic rash managed with dexamethasone and promethazine; a transient fever during the first hospitalization resolved spontaneously; and steroid-related adverse events during the second admission (generalized pruritic rash consistent with steroid-induced eczema, hypokalemia, transient psychomotor agitation, mild anemia and hypoalbuminemia) were managed symptomatically. Whether the patient remained on a corticosteroid taper at the three-month follow-up was not documented.

### 2.4 Differential diagnosis

The two-month progressive course before the second admission initially raised concern for chronic inflammatory demyelinating polyneuropathy (CIDP). Several features, however, favored an acute GBS-spectrum disorder: (1) acute-on-subacute progression with rapid worsening over four days followed by stabilization; (2) pure axonal (AMAN) electrophysiology without demyelinating features (14); (3) anti-GD1a and anti-GQ1b seropositivity characteristic of the GBS spectrum (2, 3); and (4) CSF oligoclonal bands, which are uncommon in CIDP. We nevertheless acknowledge important caveats. First, a two-month progressive phase exceeds the 4-week limit of the classic GBS definition and the Brighton criteria (15), and acute-onset CIDP (progressive phase <8 weeks) therefore remains a formal differential diagnosis (16). Second, the response to treatment cannot discriminate between these entities, because corticosteroids are a first-line treatment for CIDP but are not indicated in classic GBS (17). Third, a monophasic course without prior episodes does not by itself distinguish a first attack of CIDP from GBS (16). In the first episode, the mild CSF pleocytosis (14 cells/μL) was atypical of classic GBS, and alternative diagnoses—acute disseminated encephalomyelitis, CNS demyelinating disease, myelopathy, vasculitis and paraneoplastic disease—were actively considered and excluded by contrast-enhanced MRI, EEG, CSF and serological screening. Given the prominent upper motor neuron signs, an autoimmune myeloneuropathy or encephalomyeloneuropathy responsive to corticosteroids, and leptomeningeal disease involving the nerve roots (which may not enhance on MRI), were also considered as alternative explanations; CSF smear microscopy was normal, CSF flow cytometry was not performed, and no evidence of leptomeningeal disease was identified. A definitive distinction between GBS-spectrum disease and acute-onset CIDP is not always possible at first presentation; based on the combination of serological, electrophysiological and CSF findings, we classified the patient as having anti-GQ1b- and anti-GD1a-positive GBS–MFS–BBE overlap with AMAN electrophysiology and CSF evidence of intrathecal IgG synthesis, while acknowledging residual diagnostic uncertainty. In keeping with the Odaka criteria for BBE (external ophthalmoplegia and ataxia, with or without impaired consciousness) (18), the second episode fulfilled two elements of the triad (ophthalmoplegia and ataxia) without consciousness disturbance—an incomplete (“BBE overlap”) phenotype; comparable overlaps have been reported previously (19), and the brainstem auditory evoked potential abnormality in the first episode further supported brainstem involvement.

Final diagnosis: Anti-GQ1b- and anti-GD1a-positive GBS–MFS–BBE overlap syndrome, diagnosed mainly on the basis of the clinical course and electrophysiological findings and supported by serology and CSF analysis, with CSF evidence of intrathecal IgG synthesis indicating active CNS immune involvement (with the caveats discussed above).

### 2.5 Treatment summary

Across the two episodes, the patient received plasma exchange (three sessions of 1600 mL on 3, 4 and 7 April 2024), intravenous methylprednisolone (1 g/day in both episodes, with stepwise tapering), IVIG (27.5 g/day on 8–11 April; 27.5 g/day for five days on 11–15 May) and oral prednisone with gradual taper. We note that corticosteroids are not recommended in classic GBS (17); their use here was an off-label, hypothesis-driven choice in the context of suspected CNS involvement, and their benefit is unproven. The evidence base for the optimal treatment of MFS–BBE overlap remains limited (20).

## 3 Discussion

### 3.1 Intrathecal IgG synthesis as a CNS biomarker: what this case can and cannot show

Albuminocytologic dissociation—elevated CSF protein with normal cell counts—has long been considered a hallmark of GBS-spectrum disorders. However, this finding reflects blood-brain/blood-nerve barrier dysfunction rather than active CNS inflammation, and it is observed across the entire anti-GQ1b spectrum regardless of CNS involvement (3, 5). In contrast, intrathecal IgG synthesis represents compartmentalized, antigen-driven B-cell activation within the CNS (9–12) and can be assessed through three complementary measures: (1) an IgG index >0.70, indicating disproportionate IgG in CSF relative to albumin; (2) an IgG synthesis rate above the laboratory reference, quantifying the magnitude of the CNS immune response; and (3) CSF oligoclonal bands, the most specific marker of intrathecal synthesis (11, 12). Our patient demonstrated all three in the second episode, with a synthesis rate approximately 21 times the upper reference limit of the reporting laboratory.

Two methodological caveats should be noted. First, in the presence of severe blood–CSF barrier dysfunction—as in our patient (CSF protein 2.21 g/L; CSF albumin 1070 mg/L)—the linear IgG index and synthesis rate can overestimate intrathecal synthesis; quotient analysis using the Reiber hyperbolic function is the reference method in this setting (10). The elevated QAlb and QIgG quotients in our patient confirmed both severe barrier dysfunction and intrathecal immunoglobulin production, and the type III OCB pattern (CSF-specific bands with homologous serum bands) is consistent with intrathecal synthesis coexisting with barrier disruption. Second, IVIG administered before CSF sampling can raise CSF IgG and mimic intrathecal synthesis; in our patient, the lumbar puncture of 10 May 2024 was performed before the resumption of IVIG on 11 May.

Third, intrathecal IgG synthesis is not specific to GBS or anti-GQ1b syndromes: it occurs in most CNS immune-mediated disorders (9, 11) and can be influenced by severe blood–CSF barrier disruption and systemic immune activation, so it should be regarded as supportive rather than diagnostic. Fourth, the degree of CSF protein elevation in this patient (2.21 g/L) markedly exceeds the mean reported in GBS (approximately 113.8 mg/dL in a large case series (21)) and is more characteristic of nodopathies and paranodopathies, in which very high CSF protein is typical; the EAN/PNS 2021 CIDP guideline recommends nodal/paranodal antibody testing in this situation (16). These antibodies were not measured, which we acknowledge as a limitation.

Finally, CNS involvement does not automatically mandate treatment escalation: in the largest comparative series of Miller Fisher syndrome, IVIG was not associated with better overall outcomes than plasma exchange or supportive care, although it modestly hastened recovery of ophthalmoplegia and ataxia (22). The evidence base for intensified immunotherapy in GBS-spectrum patients with suspected CNS involvement therefore remains very limited.

We also state what this case can and cannot demonstrate. In the second episode, upper motor neuron signs (hyperreflexia, extensor plantar responses) together with ophthalmoplegia and ataxia already indicated CNS involvement on clinical grounds; the CSF markers therefore did not “unmask” clinically silent CNS disease, but provided an objective, quantifiable correlate of compartmentalized intrathecal immune activity. Their incremental diagnostic value is most plausible in patients with equivocal or absent central signs—a hypothesis that requires prospective testing. Likewise, whether intrathecal synthesis distinguishes CNS-predominant from purely peripheral phenotypes, and whether it predicts outcome, remains to be established; in our patient, marked intrathecal synthesis co-segregated with incomplete resolution of central deficits, but this association is anecdotal. Finally, the chronology should be noted: in the first episode, the intensified regimen (plasma exchange, corticosteroids, IVIG) was initiated on clinical grounds before any CSF immunology results were available, whereas in the second episode the CSF findings supported the decision to resume immunotherapy. Because the biomarker and the intensification were not temporally aligned in the first episode, no causal inference about biomarker-guided treatment is possible from this case alone.

### 3.2 Immunopathological mechanisms and the GBS–MFS–BBE continuum

The anti-GQ1b antibody syndrome spectrum represents a continuum of neuroimmune injury whose anatomical distribution depends on several factors (1–4, 18, 23). First, antibody effector functions determine pathogenicity: IgG subclass distribution (particularly IgG1 and IgG3) and complement-fixing capacity influence the extent of neural injury, as demonstrated in mouse models in which complement inhibition prevented antibody-mediated neuropathy (24, 25). Second, blood-brain and blood-nerve barrier disruption allows autoantibodies and immune effectors to reach CNS structures (26, 27). Our patient’s extremely high CSF protein (2.21 g/L) and elevated albumin quotient confirm severe barrier dysfunction facilitating antibody entry into the CNS. Third, coexistent anti-ganglioside antibodies may act on different epitopes together. Antibodies against ganglioside complexes (e.g., GQ1b–GD1a heteromers) may show enhanced pathogenicity (23, 28); this is speculative in the present case, however, because complex antibodies were not tested. Anti-GD1a is classically associated with AMAN and severe axonal injury (29–31), whereas anti-GQ1b targets oculomotor terminals and brainstem GQ1b-rich structures (32). The positive anti-sulfatide IgG is of uncertain significance; anti-sulfatide antibodies are reported in various peripheral neuropathies and are non-specific. The brainstem auditory evoked potential abnormality recorded in the first episode is consistent with the clinically evident brainstem involvement.

### 3.3 A hypothesis-generating, biomarker-guided treatment framework

Standard treatment for GBS (IVIG or plasma exchange) was established through trials that largely excluded patients with CNS involvement or BBE overlap (6, 8). The optimal management of overlap syndromes with CNS features therefore remains empiric (20). Because IVIG crosses the blood-brain barrier only to a limited degree—CSF IgG concentrations increase by a mean of ~55% after infusion but remain a small fraction of serum levels (33)—whereas corticosteroids penetrate the CNS readily, we hypothesized that adding corticosteroids to IVIG might benefit patients with evidence of intrathecal synthesis. This hypothesis is mechanistically plausible but unproven: corticosteroids are ineffective, and possibly harmful, in classic GBS (17, 34), and no randomized or controlled data support their use in CNS-predominant anti-GQ1b syndromes. We therefore present **Table 2** as a hypothesis-generating proposal intended for future investigation, not for immediate clinical application, and not as a treatment recommendation. Thresholds for “marked” intrathecal synthesis are not defined, and the framework requires prospective validation before any clinical application.

**Table 2:** Proposed biomarker-guided treatment framework (hypothesis-generating; unvalidated).

Tier	Clinical phenotype	CSF profile	Proposed approach
1	Peripheral-predominant GBS/MFS; no CNS signs	Albuminocytologic dissociation only; no intrathecal synthesis markers	Standard IVIG (0.4 g/kg/day × 5 days) or plasma exchange
2	Overlap phenotype with CNS signs	Elevated IgG index (>0.70) and/or synthesis rate; CSF OCBs	IVIG (0.4 g/kg/day × 5 days) + methylprednisolone (1 g/day × 3–5 days, then oral taper)
3	Severe/refractory, or deterioration despite Tier 2	Persistent marked intrathecal synthesis	Consider repeat IVIG, plasma exchange, or B-cell-directed therapy (e.g., rituximab) in experienced centers or clinical trials

Rationale: Tier 1—no evidence of compartmentalized CNS inflammation; Tier 2—hypothesis that corticosteroids add a CNS-penetrant anti-inflammatory effect (unproven); Tier 3—target intrathecal B-cell activity (no direct evidence).

This framework is derived from a single case and mechanistic reasoning. Thresholds for “marked” synthesis are not defined; corticosteroids are not indicated for classic GBS (17); no tier of this framework constitutes a treatment recommendation without prospective validation.

### 3.4 Limitations and future directions

This report has important limitations. (1) It is a single case without a comparator; we cannot determine whether the favorable motor recovery reflected combined therapy, IVIG alone, plasma exchange, or spontaneous improvement. (2) The distinction from acute-onset CIDP cannot be made with certainty (Section 2.4), and because corticosteroids are first-line therapy in CIDP, the treatment response does not clarify the diagnosis. (3) Although a repeat lumbar puncture on 9 June 2024 confirmed persistent protein elevation with a normal cell count, CSF immunoglobulin indices were not re-measured, so the dynamics of intrathecal synthesis during recovery remain unknown. (4) Linear measures of intrathecal synthesis may be biased by the severe barrier dysfunction; serum IgG and albumin values were not available for calculation of the Reiber-based intrathecal IgG fraction. (5) Anti-ganglioside antibody results were obtained on a single commercial platform and were not independently validated; ganglioside-complex antibodies were not tested. (6) Because CNS involvement was already clinically evident in the second episode, the incremental diagnostic value of the CSF markers could not be demonstrated here. (7) The first-episode CSF showed mild pleocytosis rather than classic albuminocytologic dissociation, which contributed to the initial diagnostic uncertainty.

Additional limitations concern the diagnostic work-up: nodal and paranodal antibodies were not tested despite the markedly elevated CSF protein (16); CSF smear microscopy was normal but CSF flow cytometry was not performed; the first-episode needle EMG was limited to a single muscle (patient declined further sampling); the reduced left median CMAP could in part reflect concurrent median neuropathy at the wrist, and the interpretation relied on the overall neurogenic pattern; and the corticosteroid status at the three-month follow-up was not documented.

Future research priorities include: (1) multicenter prospective studies with systematic CSF immunological profiling—including Reiber quotient analysis and isoelectric focusing—at baseline and follow-up, alongside serial clinical and functional assessments; (2) randomized or rigorously controlled comparisons of IVIG monotherapy versus IVIG plus corticosteroids in patients with documented intrathecal synthesis; (3) investigation of emerging biomarkers such as neurofilament light chain and anti-ganglioside-complex antibodies (23, 35); and (4) mechanistic studies to determine whether intrathecal B-cell clones expand locally or derive from peripheral trafficking. Standardization of CSF immunological assessment and the development of consensus guidelines will facilitate clinical implementation. This report was prepared in accordance with the CARE case-report guidelines (36).

## 4 Conclusion

This case demonstrates that intrathecal IgG synthesis—evidenced by an elevated IgG index (1.13), a markedly increased synthesis rate (147.10 mg/24h) and CSF oligoclonal bands—can coexist with classic albuminocytologic dissociation in an anti-GQ1b-positive GBS–MFS–BBE overlap phenotype, and that these markers may provide an objective correlate of compartmentalized CNS immune activity. We propose, as a hypothesis to be tested prospectively, that CSF immunological profiling might inform treatment intensity in anti-GQ1b syndromes with suspected CNS involvement, and that evidence of intrathecal synthesis could prompt consideration of combined IVIG and corticosteroid therapy. Given the diagnostic uncertainties and the lack of evidence for corticosteroids in classic GBS, prospective validation is required before any treatment algorithm can be recommended.

## Data Availability

De-identified clinical data underlying this case report are available from the corresponding author upon reasonable request, subject to institutional ethics and confidentiality requirements.
