# Supplementary material for: Intrathecal IgG synthesis as a biomarker for CNS involvement in anti-GQ1b antibody syndrome: a case report and hypothesis-generating treatment framework
Source: Front Immunol. 2026 Sep 1;17:1794662. doi: 10.3389/fimmu.2026.1794662 (PMC13575737; doi:10.3389/fimmu.2026.1794662)
Supplement: Supplementary file 1 [file Table1.docx]

**Supplementary Table S1. Timeline of clinical events, investigations and treatment**

*Anti-GQ1b antibody syndrome with GBS–MFS–BBE overlap phenotype and intrathecal IgG synthesis (Manuscript 1794662; updated per complete hospital records)*

| **Date** | **Category** | **Key clinical data** |
| --- | --- | --- |
| Mid-March 2024 (≈2 weeks before first admission) | Symptoms | Episodic dizziness ("cotton-wool" sensation; each episode ~30 s, recurring many times daily) and transient stabbing ear pain; subsequent numbness spreading from right upper arm to right trunk and left limbs; progressive generalized weakness (difficulty raising the head, speaking and getting out of bed); transient hypotension (60/30 mmHg). No preceding infection. |
| 27 March 2024 | Admission 1 — examination | Alert, effortful dysarthric speech; bilateral eyelid weakness; horizontal nystagmus with full range of eye movements; reduced gag reflex; MRC grade 4–5/5 weakness (right upper limb 4/5, elsewhere 5−/5); depressed tendon reflexes (especially ankle jerks); questionably positive left Babinski sign; mildly impaired finger-to-nose testing; reduced pinprick sensation on the right side. |
| 27–29 March 2024 | Laboratory screening | Routine tests, ECG, extensive screening unremarkable except mildly elevated ESR (30 mm/h), polyclonal hypergammaglobulinemia (γ-globulin 23.1%), elevated D-dimer (2.24–2.62 mg/L FEU) and mildly reduced serum sodium. ANA, ANCA, ASO, RF, HIV, syphilis, hepatitis B/C serology, HSV-1/2 DNA, CMV DNA, SARS-CoV-2 and respiratory viruses negative. |
| 28 March 2024 | Imaging | Brain MRI: minor non-specific T2 hyperintensities in both frontoparietal regions (Fazekas grade I); head-and-neck MRA without stenosis. |
| 29 March 2024 | Serology | Serum anti-ganglioside antibodies: anti-GD1a IgG positive. |
| 29 March 2024 | CSF (1st lumbar puncture) | Opening pressure 150 mmH₂O; protein 0.67 g/L (reference <0.45); total cell count 108×10⁶/L, nucleated cells 14/μL (Pandy weakly positive); glucose and chloride normal; cultures and smears negative. CSF immunoglobulins and oligoclonal bands not measured. |
| 29 March 2024 | Neurophysiology | BAEP: reduced electrical activity in the left upper pons; SSEP and VEP normal. Needle EMG limited to the left abductor digiti minimi (patient declined further sampling): no definite neurogenic or myogenic abnormality. |
| 3, 4, 7 April 2024 | Treatment — plasma exchange | Three sessions of plasma exchange (1600 mL each; femoral venous catheter placed 1 April). Two sessions complicated by transient pruritic rash, treated with dexamethasone and promethazine. |
| 7 April 2024 | Treatment — corticosteroids; CSF (2nd lumbar puncture) | Intravenous methylprednisolone 1 g/day started (reduced to 500 mg/day on 12 April). Repeat LP: pressure 80 mmH₂O; protein 1.23 g/L, glucose 4.0 mmol/L, nucleated cells 19/μL (total 1029×10⁶/L, likely blood admixture); HSV-1/2 DNA and cryptococcal antigen negative; cultures negative. |
| 8–11 April 2024 | Treatment — IVIG | IVIG 27.5 g/day (550 mL) on four consecutive days; no infusion reactions. |
| 12 April 2024 onwards | Treatment — oral corticosteroids | Oral prednisone 60 mg/day with gradual taper. |
| 15 April 2024 | Discharge 1 | Discharged with modest improvement; residual limb numbness; unable to walk independently. |
| Late April–early May 2024 | Course between episodes | Patient discontinued all immunotherapy herself; weakness and numbness recurred and worsened acutely over four days, with new-onset intermittent choking on drinking. |
| 9 May 2024 | Admission 2 — examination | Moderate dysarthria; bilateral ophthalmoplegia with horizontal nystagmus; bilateral eyelid weakness; increased limb tone (right MRC 4/5, left 3/5); brisk (3+) tendon reflexes; bilateral extensor plantar responses; limb ataxia, unable to walk independently. Admission labs: hypokalemia (K 3.1–3.4 mmol/L), albumin 32 g/L, D-dimer 2.62 mg/L FEU. |
| 10 May 2024 | CSF (3rd lumbar puncture) — before resuming immunotherapy | Opening pressure ~110 mmH₂O; protein 2.21 g/L (reference <0.45); total cell count 40×10⁶/L with normal nucleated cell count (reference 0–8×10⁶/L); glucose 3.44 mmol/L; chloride 125 mmol/L — albuminocytologic dissociation. CSF immunology: IgG index 1.13 (reference ≤0.70); intrathecal IgG synthesis rate 147.10 mg/24h (reference <7 mg/24h); oligoclonal bands type III (CSF-specific bands with homologous serum bands); CSF albumin 1070.00 mg/L, CSF IgG 47.00, IgA 53.10, IgM 92.00 mg/L; QAlb, QIgG, QIgA, QIgM quotients elevated. CSF smear microscopy normal. |
| 10 May 2024 | Serology (2nd admission) | Anti-GQ1b IgG strongly positive; anti-sulfatide IgG positive; anti-GD1a IgG positive. AQP4, MOG, GFAP, MBP and autoimmune encephalitis panel (NMDA, AMPA1/2, LGI1, GABAB, CASPR2) negative in CSF and serum. |
| 11 May 2024 | EEG | Video-EEG normal (no epileptiform activity). |
| 11–15 May 2024 | Treatment — IVIG and corticosteroids | IVIG 27.5 g/day for five days (11–15 May); intravenous methylprednisolone 1 g/day from 13 May (reduced to 500 mg/day on 15 May, then stepwise to 80 mg before conversion to oral prednisone 55–60 mg/day with weekly 5 mg tapering). Steroid-related adverse events: pruritic rash (steroid-induced eczema), hypokalemia, transient psychomotor agitation, mild anemia and hypoalbuminemia — managed symptomatically. |
| 13 May 2024 | Imaging | Chest CT: no pulmonary lesions; thyroid nodule (right lobe, ~11 mm); small hepatic cyst. |
| 14 May 2024 | Vascular ultrasound & ECG | Lower-limb arterial and deep venous ultrasound normal (no thrombosis); abdominal ultrasound: small hepatic cyst; ECG: sinus rhythm, no ST-T abnormality. |
| 15 May 2024 | Neurophysiology (2nd admission) | NCS: sensory conduction normal; markedly reduced CMAP amplitudes of bilateral median, ulnar and right peroneal motor nerves (left > right); reduced ulnar F-wave persistence; needle EMG: fibrillation potentials and positive sharp waves in bilateral tibialis anterior and abductor pollicis brevis and left biceps, normal motor unit parameters with reduced recruitment — AMAN pattern. |
| 9 June 2024 | Repeat lumbar puncture | Persistent protein elevation (Pandy 1+); total cell count 40×10⁶/L, nucleated cells 6×10⁶/L (normal). CSF immunoglobulin indices not re-measured. |
| 13 June 2024 | Discharge 2 | Discharged with improved strength (MRC 4/5 all limbs) but unable to walk independently; plantar responses converted to normal; residual dysarthria, ataxia and intermittent choking; transferred for ongoing rehabilitation. |
| ≈3 months after discharge (exact date not specified) | Follow-up | MRC 5/5 upper limbs, 4+/5 lower limbs; living independently and working. Residual central deficits: mild dysarthria (improved but not fully resolved), subtle truncal ataxia, occasional diplopia with sustained lateral gaze. Patient perspective: progressive recovery of daily function and work capacity; gradual decrease in psychological burden; returned to work. |
| Follow-up (ongoing gap) | Not documented / limitations | CSF immunoglobulin indices not re-measured at the 9 June LP (limits biomarker–recovery correlation). Corticosteroid taper status at the three-month follow-up not documented. Nodal/paranodal antibodies not tested. CSF flow cytometry not performed. |

Abbreviations: AMAN, acute motor axonal neuropathy; ANA, antinuclear antibody; ANCA, antineutrophil cytoplasmic antibody; ASO, antistreptolysin O; BAEP, brainstem auditory evoked potentials; BBE, Bickerstaff brainstem encephalitis; CMV, cytomegalovirus; CSF, cerebrospinal fluid; EEG, electroencephalography; EMG, electromyography; ESR, erythrocyte sedimentation rate; GBS, Guillain-Barré syndrome; HSV, herpes simplex virus; IVIG, intravenous immunoglobulin; LP, lumbar puncture; MFS, Miller Fisher syndrome; MRC, Medical Research Council; MRI, magnetic resonance imaging; NCS, nerve conduction studies; PSW, positive sharp waves; SSEP, somatosensory evoked potentials; VEP, visual evoked potentials.
